# The Effect of High Polyphenol Extra Virgin Olive Oil on Blood Pressure and Arterial Stiffness in Healthy Australian Adults: A Randomized, Controlled, Cross-Over Study

**DOI:** 10.3390/nu12082272

**Published:** 2020-07-29

**Authors:** Katerina Sarapis, Colleen J. Thomas, Johanna Hoskin, Elena S. George, Wolfgang Marx, Hannah L. Mayr, Greg Kennedy, Andrew Pipingas, Jane C. Willcox, Luke A. Prendergast, Catherine Itsiopoulos, George Moschonis

**Affiliations:** 1 Department of Dietetics, Nutrition and Sport, School of Allied Health, Human Services and Sport, La Trobe University, Melbourne 3086, Australia; k.sarapis@latrobe.edu.au (K.S.); 19974176@students.latrobe.edu.au (J.H.); elena.george@deakin.edu.au (E.S.G.); wolf.marx@deakin.edu.au (W.M.); J.Willcox@latrobe.edu.au (J.C.W.); 2 Department of Physiology, Anatomy and Microbiology, School of Life Sciences, La Trobe University, Melbourne 3086, Australia; colleen.thomas@latrobe.edu.au; 3 Institute for Physical Activity and Nutrition (IPAN), School of Exercise and Nutrition Sciences, Deakin University, Geelong 3220, Australia; 4 Impact (the Institute for Mental and Physical Health and Clinical Translation), Food & Mood Centre, Deakin University, Geelong 3220, Australia; 5 School of Allied Health, Human Services and Sport, La Trobe University, Melbourne 3086, Australia; hmayr@bond.edu.au (H.L.M.); catherine.itsiopoulos@murdoch.edu.au (C.I.); 6 Bond University Nutrition and Dietetics Research Group, Faculty of Health Sciences and Medicine, Bond University, Brisbane 4226, Australia; 7 Department of Nutrition and Dietetics, Princess Alexandra Hospital, Brisbane 4102, Australia; 8 Centre for Human Psychopharmacology, Swinburne University of Technology, Melbourne 3122, Australia; gikennedy@swin.edu.au (G.K.); apipingas@swin.edu.au (A.P.); 9 Department of Mathematics and Statistics, School of Engineering and Mathematical Sciences, La Trobe University, Melbourne 3086, Australia; luke.prendergast@latrobe.edu.au; 10 College of Science, Health, Engineering and Education, Murdoch University, Perth 6150, Australia

**Keywords:** olive oil, extra virgin olive oil, polyphenols, cardiovascular disease, blood pressure, hypertension, arterial stiffness

## Abstract

Extra virgin olive oil (EVOO) is suggested to be cardioprotective, partly due to its high phenolic content. We investigated the effect of extra virgin high polyphenol olive oil (HPOO) versus low polyphenol olive oil (LPOO) on blood pressure (BP) and arterial stiffness in healthy Australian adults. In a double-blind, randomized, controlled cross-over trial, 50 participants (age 38.5 ± 13.9 years, 66% female) were randomized to consume 60 mL/day of either HPOO (360 mg/kg polyphenols) or LPOO (86 mg/kg polyphenols) for three weeks. Following a two-week washout period, participants crossed over to consume the alternate oil. Anthropometric data, peripheral BP, central BP and arterial stiffness were measured at baseline and follow up. No significant differences were observed in the changes from baseline to follow up between the two treatments. However, a significant decrease in peripheral and central systolic BP (SBP) by 2.5 mmHg (95% CI: −4.7 to −0.3) and 2.7 mmHg (95% CI: −4.7 to −0.6), respectively, was observed after HPOO consumption. Neither olive oil changed diastolic BP (DBP) or measures of arterial stiffness. The reductions in SBP after HPOO consumption provide evidence for a potentially widely accessible dietary intervention to prevent cardiovascular disease in a multiethnic population. Longer intervention studies and/or higher doses of EVOO polyphenols are warranted to elucidate the potential effect on DBP and arterial stiffness.

## 1. Introduction

Cardiovascular disease (CVD) is the leading cause of mortality and morbidity worldwide. Established risk factors such as hypertension, dyslipidaemia, diabetes and obesity contribute to 9.7 million annual deaths related to CVD globally [1]. The most recent Australian data, collected between 2017 and 2018, indicates 27% of all deaths (43,477 deaths) were attributed to CVD [2]. A similarly high proportion of Australian adults (~34%) have diagnosed hypertension [3]. Previous studies have indicated that changes in peripheral and central hemodynamics such as in peripheral (brachial) and central (aortic) BP and pressure wave reflections contribute to the development of adverse cardiovascular events [4]. Moreover, stiffening of the central elastic arteries, such as the aorta and the pulmonary arteries, is an accepted independent predictor of CVD risk and is positively associated with systolic hypertension [5,6]. Several surrogate markers reflecting vascular health are used in clinical practice. Pulse wave velocity (PWV), estimated by non-invasive applanation tonometry and pulse wave analysis, is considered the gold-standard marker of arterial stiffness [7]. Furthermore, systemic arterial wave reflections, as measured by the augmentation index (AIx), provide additional clinical information on CVD, while assessment of central BP and pulse pressure (PP) provides further predictive value beyond the corresponding brachial BP [6,8,9].

Extensive evidence indicates that certain dietary patterns are cardioprotective [10]. The traditional Mediterranean diet (MedDiet), has been shown to improve CVD risk factors including lipidemic and glycaemic profile, markers of inflammation and oxidative stress [11,12]. The MedDiet is plant rich, with staple foods consisting of wholegrain cereals, vegetables, fresh fruit, seafood, legumes, nuts and red wine [12,13]. Previous studies have demonstrated that MedDiet food components improve vascular health. Moderate consumption of red wine has been found to reduce BP and improve arterial stiffness in healthy individuals [14] and patients with coronary artery disease [15,16]. Furthermore, regular consumption of olive oil (OO), which is the principal source of dietary fat in the MedDiet, has demonstrated BP-lowering effects [17,18]. 

The reported cardioprotective benefits of OO have been mostly attributed to the presence of variable concentrations of bioactive compounds, including polyphenols (also referred to as biophenols), mainly known for their anti-inflammatory and antioxidant properties [12,19,20]. One of the determinants of final OO polyphenol concentration is the oil extraction procedure [20,21,22]. In particular, extra virgin olive oil (EVOO) is obtained by mechanical extraction techniques under conditions that preserve high polyphenol concentrations, whereas refined OO (ROO) is subject to both physical and/or chemical processing, which significantly lowers the phenolic content [22]. Although there is some evidence linking dietary polyphenol intake, including those in virgin OOs, with decreased CVD risk [23,24], this favourable effect of polyphenols is not currently taken into consideration by dietary guidelines, thus indicating a need for further relevant evidence. 

Our research team recently conducted a meta-analysis of the published literature to determine the effects of HPOO consumption, compared with LPOO, on cardiovascular markers [19]. This meta-analysis indicated that HPOO can improve outcomes related to cholesterol (total and high-density lipoprotein (HDL)) and oxidative stress (oxidized low-density lipoprotein (LDL) and malondialdehyde) compared to the LPOO intervention arm. However, no significant changes were observed with respect to SBP and DBP after either OO consumption (daily dose ranged between 25 and 75 mL), while none of the included studies reported measures of arterial stiffness. In 2019, a network meta-analysis reported that EVOO may reduce oxidized LDL (ox-LDL) and LDL cholesterol compared to ROO and LPOO, respectively, while a dose–response relationship was observed between higher intakes of OO phenolic compounds and lower SBP and ox-LDL values [25]. As also stated in another recent review, most intervention studies investigating the effect of HPOO on CVD risk markers have been conducted in Mediterranean populations that have high habitual OO intake [26], thus highlighting the need for additional research on multiethnic populations with different habitual food cultures. Hence, the aim of the current study was to examine the effect of daily consumption of (60 mL) raw extra virgin HPOO, compared to LPOO, for 3 weeks, on peripheral and central BP and arterial stiffness in Australian adults with no previously diagnosed medical condition.

## 2. Materials and Methods 

### 2.1. Study Population

The OLIVAUS study [20] was conducted according to the Guidelines for Good Clinical Practice (GCP), the guidelines laid down in the Declaration of Helsinki and the CONSORT reporting guidelines. All procedures involving human subjects were approved by the Human Research Ethics Committee of La Trobe University (HEC17-067) and written informed consent was obtained from all volunteers. The trial protocol has been registered with the Australia New Zealand Clinical Trials Registry ACTRN12618000706279.

All participants were recruited in Melbourne, Australia, via social media and La Trobe University email database advertising, word of mouth and posters on campus. A standardized screening procedure was followed in order to identify eligible participants, who were required to be within the age range of 18–75 years and a body mass index (BMI) of 18.5–40 kg/m^2^. Exclusion criteria included non-English-speaking individuals, pregnant or lactating women, smokers, individuals on a special type of diet for medical reasons (e.g., gluten free for coeliac disease) and/or with a high habitual OO intake (>1 tablespoon/day). Exclusion also applied if individuals were taking vitamins or antioxidant supplements as part of a regular regime and were unable to discontinue their use for the duration of the trial (with the exception of iron, calcium and Vitamin D). Finally, study subjects taking prescribed medication (e.g., antihypertensive agents, lipid-lowering drugs, non-steroidal ant-inflammatory drugs) and those with diagnosed chronic diseases (diabetes, hyperlipidaemia, hypertension, and inflammatory conditions), gut-related diseases or any other condition that could impair adherence were also excluded.

### 2.2. Study Design and Procedure

The OLIVAUS study was a double-blind, cross-over, randomized controlled trial (RCT) aiming to evaluate the effect of extra virgin HPOO consumption on CVD risk markers in comparison with a commercially available OO which was low in polyphenols (LPOO). Prior to the main study, a pilot study was conducted with five study participants in order to test the feasibility of the study protocol and the data collection tools [27]. Enrolled participants were randomly assigned, in a 1:1 ratio, to one of two treatment arms, i.e., Group (1) extra virgin HPOO/LPOO or Group (2) LPOO/extra virgin HPOO, using the block-randomization method of a software program for sequence. Blocks of 6 participants were generated by a senior researcher, who was not directly involved in the participant recruitment or data collection phase. Allocation of each participant was emailed to the research team at the commencement of the study by a researcher who was not involved in any participant contact.

Study participants were requested to consume a daily dose of 60 mL of either type of raw OO over 2 intervention periods of 3 weeks each, in conjunction with their habitual diet. The two types of OO varied only in their phenolic content (i.e., 360 mg/kg in HPOO vs. 86 mg/kg in LPOO) but did not differ with respect to the rest of their nutrient composition, including their fatty acid profile. Two washout periods, of 2 weeks each, during which study participants were instructed to avoid olives and OO consumption, preceded the first and the second intervention periods of OO administration. The intervention in the present study was designed with a daily dose of 60 mL OO, which reflects the habitual intake in populations where the cardioprotective benefits of virgin OO have been previously reported [19,25,26].

Participants were provided with OO bottles at the beginning of each intervention period. The OOs were supplied in dark coloured glass containers to minimise phenolic content loss due to sunlight. To ensure blinding of the researchers to the OO type, each bottle was assigned a different code number that was concealed from study participants and research team members. This was disclosed only after the completion of the statistical analyses. To assess the level of adherence to the intervention, participants were instructed to return the containers at the end of each intervention period so that the daily amount of unconsumed OO could be measured and recorded. Study participants were also instructed to keep a written record of daily OO consumed during each intervention period using a checklist provided to them. This information was recorded by research team members after the end of each intervention period. Full details of the study protocol, including a comparison of the concentrations of total polyphenols and polyphenol subclasses in each of the two types of OOs, are provided elsewhere [20].

### 2.3. Measurements

#### 2.3.1. Socio-Demographics, Use of Medication and Dietary Supplements

Socio-demographic data were collected from eligible participants during a scheduled interview at our trial clinic room located at La Trobe University. Trained researchers conducted all interviews using a standardized questionnaire. Specifically, the socio-demographic data collected during this interview included age, gender, language(s) spoken at home, level of education, ethnicity and parental country of birth. Any medications and dietary supplements taken by the study participants were also recorded.

#### 2.3.2. Dietary Intake

A 3-day food diary was used to collect information on the dietary intake of study participants during two weekdays and one weekend day (preferably non-consecutive) at baseline and follow up of each intervention period. Specifically, study participants were instructed to record details on their intake of food and beverages, including information on the quantity, type/brand and cooking methods of the consumed items. The level of detail required to be recorded in the diary as well as additional strategies on how to incorporate raw, uncooked OO in their habitual diet were provided to study participants at a pre-baseline meeting by a trained nutritionist. The completed food diaries were returned and checked by the research team members for potential wrong or missing entries during the scheduled interviews with the study participants. All dietary intake data were analyzed for energy, macro- and micronutrient content using FoodWorks^®^9 software (Xyris Software Pty Ltd., Brisbane, Queensland, Australia). 

#### 2.3.3. Physical Activity 

Physical activity (PA) was assessed using the Active Australia Survey (AAS) questionnaire [3], a tool that has been validated in the Australian population. This questionnaire is designed to assess participation in a range of leisure-time physical activities of light, moderate and vigorous intensity. The questionnaire consists of eight questions, which assess the number of sessions and total weekly time (hours and/or minutes) spent for each activity type. Study participants were required to complete and submit the AAS questionnaire during the week preceding the interviews at the first baseline and at the last follow-up meeting. The amount of time (in minutes per day) that study participants were engaged in physical activity of different intensity was calculated and used for data analysis.

#### 2.3.4. Anthropometric Measurements

Anthropometric measurements were conducted four times during the study, i.e., at baseline and follow up of each intervention period. Body weight and standing height were measured with study participants in light clothing and barefoot, using a digital scale (WM203, Willawong QLD, Australia), to the closest 0.1 kg and a wall-mounted stadiometer (SE206, Seven Hills, NSW, Australia) to the nearest 0.1 cm, respectively. Waist circumference (WC) was measured to the nearest 0.1 cm, using a flexible steel tape calibrated in cm with mm graduations (Luftkin W606PM, Sparks, MD, USA) directly over the skin at the umbilicus level. Body mass index (BMI) was calculated using Quetelet’s equation (weight (kg)/height (m)^2^). Using World Health Organization (WHO) cut-off points for BMI, study participants were classified as underweight (BMI < 18.5 kg/m^2^), normal weight (BMI 18.5–24.9 kg/m^2^), overweight (BMI 25.0–29.9 kg/m^2^) or obese (BMI ≥ 30 kg/m^2^) [28]. Furthermore, gender-specific WC cut-off points proposed by the WHO were also used to categorise study participants for CVD risk: normal (WC < 94 cm in men and <80 cm in women), high CVD risk (WC 94–102 cm in men and 80–88 cm in women) and very high CVD risk (WC > 102 cm in men and 88 cm in women) [29].

#### 2.3.5. Hemodynamic Indices

##### Blood Pressure

Peripheral (brachial) and central (aortic) blood pressure (BP) were measured using applanation tonometry with a SphygmoCor XCEL device (Model XCEL, AtCor Medical, Sydney, Australia), at baseline and follow-up examinations at each intervention period. Following a minimum of 5 min rest in the supine position, peripheral brachial systolic BP (SBP) and diastolic BP (DBP) was measured using a blood pressure cuff affixed to the upper left arm. Three consecutive BP recordings were made and the average of the last two recordings was used for data analysis. In addition, central SBP and DBP, as well as PP measures were automatically derived via the brachial BP cuff. The BP categories recommended by the American College of Cardiology (ACC)/American Heart Association (AHA) were used to classify study participants into those with Normal BP (SBP/DBP < 120/80 mmHg), Elevated BP (SBP 120–129 mmHg and DBP < 80 mmHg), Hypertension Stage I (SBP 130–139 mmHg or DBP 80–89 mmHg) and Hypertension Stage II (SBP ≥ 140 mmHg or DBP ≥ 90 mmHg) [9].

##### Arterial Stiffness

Measures of peripheral and central arterial stiffness, using pulse wave analysis (PWA) and pulse wave velocity (PWV), were obtained non-invasively with the SphygmoCor XCEL device (Model XCEL, AtCor Medical, Sydney, Australia). This was carried out using the standard procedure as outlined in our previous paper [8]. PWA is a non-invasive, valid and reliable technique to investigate mechanical properties of the arterial tree, using central blood pressures and analysis of systemic arterial wave reflection. Peripheral arterial stiffness indices of augmentation pressure (AP) and the augmentation index (AΙx) were derived automatically by the device as part of the standard BP measurement procedure. The AP was calculated as the difference between the first and second systolic peak, while the AIx was calculated as the percentage contribution that the AP makes to the overall PP (*AIx* = *AP*/*PP* × 100). PWV was measured using a tonometer to capture the carotid waveform, while a femoral cuff was placed high on the left thigh in order to capture the femoral waveform. The PWV was then calculated by dividing the distance between the carotid and femoral measurement sites by the transit time. This method is considered the gold standard technique for assessing central arterial stiffness. 

### 2.4. Sample Size Calculation 

Power calculations showed that a sample size of 40 was adequate to provide sufficient statistical power to detect a statistically significant between-group difference of 5% and a standard deviation (SD) of 11 in HDL-C efflux levels (i.e., the primary outcome of the OLIVAUS study), with 80% power and 5% level of significance [30]. The total sample size was set at 50 study participants, in order to also account for an attrition rate of 20%. Although, the selected sample was adequate for the examination of HDL-C efflux, this might not be the case for the secondary outcomes of the OLIVAUS study, including BP and measures of arterial stiffness. 

### 2.5. Statistical Analysis 

All statistical analyses were conducted using the SPSS statistical software for Windows (IBM, version 24.0; IBM, Armonk, NY, USA). For all continuous variables, the Kolmogorov–Smirnov test was performed to examine the normality of their distribution. A general linear model, i.e., repeated-measures ANOVA (analysis of variance), was used to examine the between-group differences (treatment effect, i.e., extra virgin high vs. low polyphenol OO) of mean values at each time point of measurement, the within-group changes (time effect) from baseline to follow up in each intervention arm, and the differences in the changes from baseline to follow up between the two intervention arms (treatment × time interaction effect). Both per protocol (PP) and intention-to-treat (ITT) analyses were performed. The PP analyses were conducted in study participants who had full data from baseline to follow up in the first or the second intervention period. For the ITT analyses, multiple imputations were conducted in order to compensate for all missing values. Five imputed models derived from this process. Considering that the PP and the ITT analyses provided similar results (i.e., mean values, mean changes and statistical significance), the results coming from the latter are presented in this article. In all statistical analyses, adjustments were made for gender and age. Data are presented either as the mean ± SD, as estimated marginal means and standard errors (SE) or as the mean change and 95% confidence interval of change (CI) for continuous variables and as frequency (*n*) and percentage (%) for categorical ones. All reported *p* values are two tailed, and the level of statistical significance is set at *p* < 0.05.

## 3. Results

**Figure 1:**
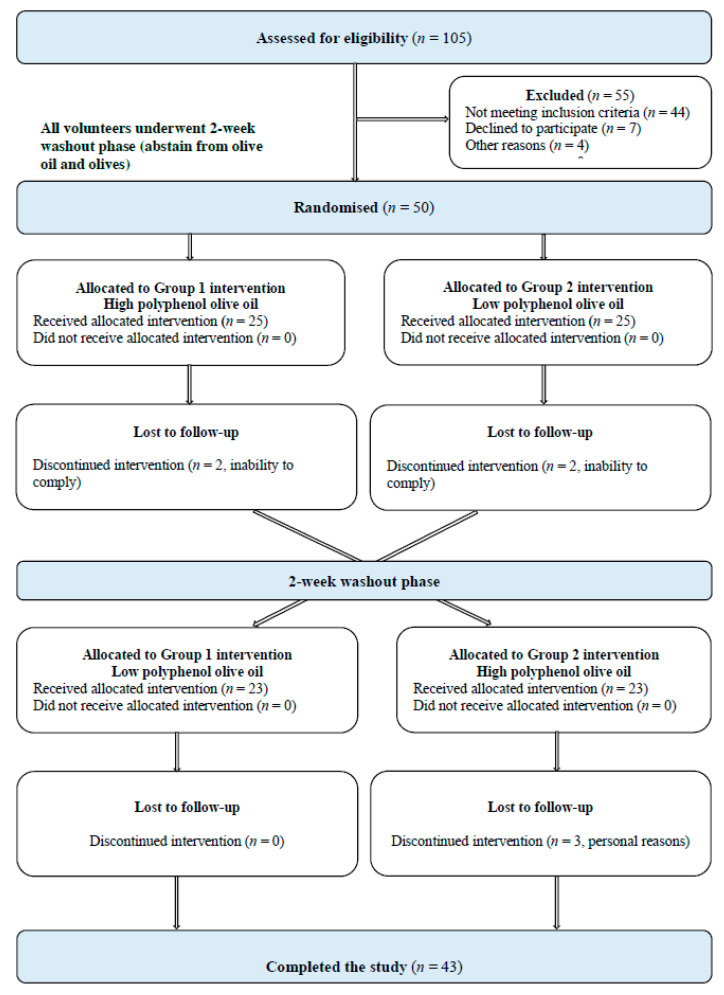
Study participant flow diagram.

Fifty volunteers (*n* = 33 females, and *n* = 17 males), from 105 interested individuals who agreed to be screened, were eligible and enrolled in the study from July 2018 through to October 2019. Seven participants discontinued the intervention, due to inability to comply (*n* = 4) and for personal reasons, (*n* = 3) and therefore 43 participants completed the study. Figure 1 provides the study participant flow diagram. Minor adverse events were recorded after the consumption of both intervention OOs, with nausea and heart burn being the most common symptoms. The proportion of participants that experienced symptoms of nausea (24%) and heart burn (6%) was comparable between the HPOO and LPOO group.

### 3.1. Baseline Characteristics of Study Participants

Table 1 presents the descriptive characteristics of study participant socio-demographics, anthropometrics and hemodynamic indices in the total sample (*n* = 50) and by gender. Study participants had a mean age of 38.5 ± 13.9 years (the age range was between 20 and 70 years) and their mean years of education was 17.3 ± 3.5. In addition, the majority of study participants were females (66%), had a tertiary education (86%) and were born in Australia (70%). No significant gender differences were observed in any of these socio-demographic characteristics. The mean BMI and WC was 24.7 ± 3.5 kg/m^2^ and 86.9 ± 11.2 cm, respectively, with no significant differences between genders. In addition, 44% of study participants were overweight and 4% were obese. Based on their WC measurements, 16% had a high cardiometabolic risk and 24% had very high risk. Although there were no significant differences between genders observed in BMI and WC, compared to females, male study participants were taller (179.3 ± 6.8 cm vs. 163.6 ± 5.8 cm, *p* < 0.001) and had a higher body weight (79.6 ± 9.6 kg vs. 66.1 ± 11.9 kg, *p* < 0.001). At baseline, the mean peripheral SBP and DBP for the cohort was 120.0 ± 13.4 and 69.9 ± 8.4 mmHg, respectively, while 18% of study participants were categorised as having elevated BP, 20% had Stage 1 Hypertension and 8% had Stage 2 Hypertension. Mean central SBP and DBP was 106.8 ± 13.3 and 70.6 ± 8.7 mmHg, respectively, mean heart rate was 61.5 ± 10.2 bpm and PWV was 9.5 ± 1.4 m/s. There were no significant differences between genders in any of these hemodynamic indices. 

### 3.2. Effect of LPOO and HPOO on Dietary Intake and Physical Activity

The changes observed in dietary energy, macro- and micronutrient intake from baseline to follow up, as well as the differences between treatment arms are summarized in Table 2. The changes from baseline to follow up were not significantly different between the two treatment arms. However, dietary energy intake increased significantly in participants following LPOO (by 1806.1 kJ/day, 95% CI: 1075.4 to 2536.8) and HPOO (by 1766.6 kJ/day, 95% CI: 1035.9 to 2497.3). Consumption of LPOO and HPOO also significantly increased intake of total fat (by 49.3 g/day, 95% CI: 41.1 to 57.4 and 46.0 g/day, 95% CI: 37.8 to 54.1, respectively), SFA (by 7.4 g/day, 95% CI: 4.0 to 10.8 and 6.5 g/day, 95% CI: 3.1 to 9.9, respectively), MUFA (by 36.8 g/day, 95% CI: 33.2 to 40.3 and by 35.1 g/day, 95% CI: 31.6 to 38.6, respectively) and PUFA (by 3.1 g/day, 95% CI: 1.0 to 5.1 and by 3.0 g/day, 95% CI: 1.0 to 5.1, respectively). In addition, no significant within-group changes or between-group differences were observed in the other examined macronutrients (protein, carbohydrates and dietary fibre), nor in micronutrients such as sodium, potassium, magnesium and calcium, including caffeine. Regarding physical activity, no within-group changes or between-group differences were observed in the time study participants were engaged in physical activities of moderate–vigorous intensity over the intervention period (data not shown). 

### 3.3. Effect of LPOO and HPOO on Anthropometrics

Table 3 summarizes the changes observed in anthropometric indices from baseline to follow up and the relevant differences between the two intervention arms. There was a small but significant increase in body weight by 0.4 kg (95% CI: 0.2 to 0.7) following the LPOO intervention, but this change was not found to differ compared to the non-significant change observed in HPOO group. No within-group changes or between-group differences were observed in BMI and WC after the daily consumption of the two intervention oils.

### 3.4. Effect of LPOO and HPOO on Peripheral BP, Central BP and Arterial Stiffness

**Figure 2:**
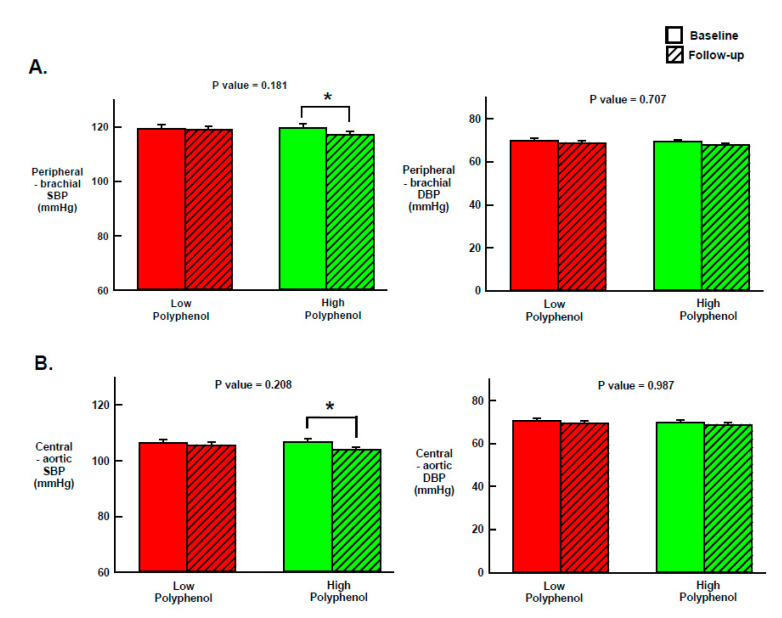
Effect of 3-weeks daily consumption of extra virgin high polyphenol olive oil (HPOO; 360 mg/kg polyphenols) and low polyphenol OO (LPOO; 86 mg/kg polyphenols) on mean peripheral (**A**) and central (**B**) blood pressure. *N* = 50 participants. SBP, systolic blood pressure; DBP, diastolic blood pressure. Data are mean±standard deviation. The P values in the Figure indicate the between-group differences in the changes from baseline to follow-up (treatment*time effect) for each blood pressure measure. The asterisk (*) indicates significance (*p* < 0.05) of within-group changes from baseline to follow-up.

The effect of the two intervention OOs on peripheral and central BP are illustrated in Figure 2. The changes from baseline to follow up were not significantly different between the two treatment arms. However, compared to baseline, peripheral (brachial) and central (aortic) SBP was significantly reduced after HPOO by 2.5 mmHg (95% CI: −4.7 to −0.3) and by 2.7 mmHg (95% CI: −4.7 to −0.6), respectively. No other significant within-group changes or between-group differences were observed in peripheral and central DBP, as well as in the rest of the examined hemodynamic (i.e., PP and HR) and arterial stiffness indices (i.e., AP, AIx and PWV) (Table 4).

### 3.5. Compliance to Treatment

Compliance to treatment was high, as reflected in the OO volume returned by participants after each intervention period. Based on the measured actual remaining OO, compliance was found to be 92% for both the LPOO and HPOO group after the first intervention period, while 92% for the LPOO group and 90% for the HPOO group after the second intervention period. Nevertheless, compliance was not found to differ significantly between the two groups (Supplementary Table S1).

## 4. Discussion

The present double-blind, cross-over, randomized controlled trial investigated the effect of daily consumption of 60 mL raw extra virgin HPOO in comparison with LPOO, each for 3 weeks, on BP and arterial stiffness in Australian adults. The key finding was that peripheral and central SBP decreased significantly by 2.5 and 2.7 mmHg, respectively, after extra virgin HPOO (phenolic content 360 mg/kg) consumption. However, no significant differences were observed between the two interventions with regards to the changes in peripheral and central SBP. No significant within-group changes or between-group differences were either observed on diastolic BP, and measures of arterial stiffness.

The significant decrease reported from our study in peripheral SBP is consistent with the limited number of RCTs that have examined the effect of HPOO consumption on peripheral BP, but after providing different doses of OO, different phenolic content of the administered oil, and varying intervention duration. In this regard, Moreno-Luna et al. [24] described that daily consumption of 60 mL HPOO with the highest phenolic content reported in the published literature (i.e., 564 mg/kg) for 8 weeks, significantly reduced peripheral SBP and DBP (by 7.9 and 6.6 mmHg, respectively) compared to ROO with no polyphenols, in young women with mild hypertension. Using an OO with high polyphenol concentration comparable to our study, Bondia-Pons et al. [31] reported that 9 weeks daily consumption of 25 mL of OO (366 mg/kg of polyphenols) significantly decreased peripheral SBP (~2.4 to 4.4 mmHg) in healthy non-Mediterranean men living in Europe. Other authors have described that 3 weeks daily consumption of 25 mL of HPOO (366 mg/kg of polyphenols) induced a significant reduction in peripheral SBP by 4.2 mmHg, through modulating the expression of genes that are related to the renin–angiotensin–aldosterone system (RAAS) [32]. In agreement with our results, the two aforementioned studies did not show any significant changes in peripheral DBP. These findings are supported by a recent meta-analysis, reporting that consumption of OOs with at least 150 mg/kg polyphenols significantly reduces peripheral SBP but not peripheral DBP [26], although there is evidence coming from one clinical trial indicating that OO with less phenolic content might also exert SBP-lowering effects. In this context, the NUTRAOLEUM study showed that daily consumption of 30 mL of virgin OO (phenolic content, 124 mg/kg) for 3 weeks significantly reduced peripheral SBP by 2.0 mmHg but not peripheral DBP, in healthy adults [33].

It is noteworthy, that other clinical trials also examining the effect of HPOO on peripheral SBP and DBP reported either significant results only for peripheral DBP or no significant findings on BP. In this regard, the EUROLIVE study demonstrated that 3 weeks of daily consumption of 25 mL EVOO, containing 366 mg/kg of polyphenols, significantly reduced peripheral DBP, but had no effect on SBP in healthy men [34], while another recent meta-analysis showed no significant pooled effect of the consumption of HPOO (150 to 800 mg/kg phenolic content) on peripheral SBP and DBP [19].

To the best of our knowledge, this is the first study reporting a significant reduction in central SBP after consumption of HPOO. This is of importance, considering that raised central BP has been positively associated with cardiovascular risk and mortality [16,35]. The effect of different bioactive nutrients (e.g., omega 3 fish oils, Vitamin C, and Vitamin E) on central hemodynamic markers (i.e., central SBP and DBP), either in the acute postprandial state or after long-term use, has been previously reported [36]. However, there is currently no evidence stemming from long-term RCTs regarding the effects of OO polyphenols alone, on these markers. Considering the scarcity of evidence and although not directly comparable with our study, Papamichael et al. [16] reported significant postprandial reductions in both central SBP and DBP, ranging from 3 to 5 mmHg, after the consumption of meals combining OO and red wine by healthy study participants. However, the combined meal design makes it difficult to give attribution to the OO and/or wine for the favourable effects observed on central BP. The study’s authors proposed that certain nutrients may exert a decrease in peripheral resistance, and consequently wave reflections and left ventricular afterload, thus resulting in a decrease in central SBP [16]. However, the mechanisms by which virgin OO minor compounds might exert their beneficial effects on central hemodynamic markers remain unclear. Therefore, further studies are warranted to reach final conclusions about the effect of OO polyphenols on central SBP and/or DBP.

Dietary intake and body weight changes observed in our study deserve comment in the context of the favourable effect of HPOO on peripheral and central SBP. In this regard, the addition of 60 mL of OO in participants’ habitual diet resulted in significant increases in caloric intake leading to weight gain in both intervention groups. Previous cross-sectional and prospective studies have reported that body weight gain is directly associated with increases in arterial BP in normotensive subjects [37], with a 1 kg increase in body weight predicting a 0.63 and 0.42 mmHg increase in SBP and DBP, respectively [38]. Based on the above, the increases in body weight of 0.2 and 0.4 kg observed in the HPOO and LPOO group, respectively, would be expected to lead to corresponding increases in BP. However, that was not the case in our study, indicating a potential counterbalancing effect of OO polyphenols on weight gain. In this regard, the phenolic content of 360 mg/Kg in HPOO, which was much higher compared with the effective threshold of 150 mg/kg reported by a recent meta-analysis [26], could provide a basis for interpreting the significant reduction in peripheral and central SBP observed in this group, despite the non-significant weight gain of 0.2 kg.

Further to dietary energy intake, our study also recorded the intake of macro- (i.e., protein and dietary fibre) and micronutrients (i.e., sodium, potassium, magnesium and calcium) that have established effects on BP levels [39,40,41,42]. In this regard, there were no differences between the two intervention arms, indicating that the only dietary factor which could account for the observed favourable effect to reduce SBP, was the higher phenolic content in HPOO compared to LPOO. However, it is not clear why the higher phenolic content in HPOO has a significant lowering effect only on SBP but not on DBP. It could be speculated that both quantity (i.e., dose) and quality (i.e., chemical structure) of polyphenols in EVOO may exert differential effects on the vascular system [43,44]. Nevertheless, further clinical trials are required to examine the effect of OOs with different phenolic profile on BP, arterial stiffness and other cardiometabolic risk markers.

To the best of our knowledge, the OLIVAUS study is the first human clinical trial to investigate the effect of OO polyphenols on measures of arterial stiffness through applanation tonometry. Stiffening of the arterial wall in the larger central arterial system represents an important CVD risk marker [6] and the early detection of such abnormalities can inform relevant preventive or treatment initiatives. However, the present study did not detect within-group changes or between-group differences in any measures of arterial stiffness after either OO intervention. The absence of significant findings may be partly attributed to our study being adequately powered for its primary outcome, while this might not be the case for the secondary outcomes, including measures of arterial stiffness. Despite the scarcity of evidence in this field, there is a large body of published literature documenting the effect of polyphenols on biochemical markers of endothelial function, which also represent other surrogate measures of arterial stiffness. In this context, Sanchez-Rodriquez et al. [33] investigated the effect of three virgin OOs enriched with polyphenols (124, 490 and 487 mg/kg) and triterpenes (86 ppm, 86 ppm and 389 ppm, respectively) on endothelial function biomarkers in healthy adults. These investigators reported significant reductions in plasma levels of the vasoconstrictor hormone endothelin-1 at the end of the three interventions and regardless of triterpene content. In another clinical trial in women with mild hypertension, daily consumption of extra virgin HPOO (564 mg/kg polyphenols) for 8 weeks significantly decreased plasma levels of asymmetrical dimethylarginine (ADMA), which is a surrogate marker of poor endothelial function [45]. The participants also had significantly increased concentrations of vasodilating nitric oxide (NO) molecule after the intervention, supporting a beneficial effect of high polyphenol OO on endothelial function [24,45].

The findings reported in our study should be interpreted in light of its strengths and limitations. The main strength of the present study is its randomized, double-blind, cross-over design that reduces interindividual variability and increases the external validity of the study findings. The use of applanation tonometry represents another strength, since it is a state-of-the-art, non-invasive method to measure BP and arterial stiffness. In addition, in a multiethnic population that is not accustomed to a high consumption of OO, our participants’ compliance was overall high throughout both intervention periods. On the other hand, one of the limitations of the present study is that the sample size was calculated on the basis of the expected differences only in its primary outcome (i.e., HDL-efflux). Another limitation could be the potential effect of seasonality on the examined outcomes, due to the fact that the participants were enrolled in the study gradually (i.e., from July 2018 through to October 2019). Lastly, despite the inclusion of a washout period before the initiation of the intervention and between the intervention periods, there is no guarantee that any potential carry-over effect on the examined hemodynamic markers was completely avoided. However, pairwise comparisons that examined potential carry-over effects were insignificant for all hemodynamic markers.

## 5. Conclusions

To our knowledge, the OLIVAUS study is the first to examine the effect of OO polyphenols on peripheral and central SBP and DBP as well as on measures of arterial stiffness in Australian adults. Although there were no significant differences between OO treatments in any of the examined outcomes, there was a significant reduction in peripheral and central SBP after daily consumption of extra virgin HPOO for 3 weeks. This provides evidence for a potentially widely accessible dietary intervention that can reduce CVD risk in a multicultural context, such as in Australia. However, additional clinical trials of longer duration and use of EVOO with different phenolic content and profile are required to shed more light on the potential effect of OO polyphenols on other CVD risk markers, including DBP and arterial stiffness.

## Figures and Tables

**Table 1:** Descriptive characteristics of study participants.

	Total Sample (*n* = 50)	Males (*n* = 17)	Females (*n* = 33)	*p*-Value *
Socio-Demographics	Mean (SD)	Mean (SD)	Mean (SD)	
Age (years)	38.5 (13.9)	33.4 (11.6)	41.2 (14.4)	0.058
Education (years)	17.3 (3.5)	17.4 (3.7)	17.2 (3.5)	0.895
**Highest Level of Education**	***n* (%)**	***n* (%)**	***n* (%)**	0.241
Secondary School	2 (4.0)	0 (0.0)	2 (6.1)	
Tertiary	43 (86.0)	17 (100.0)	26 (78.8)	
Trade	2 (4.0)	0 (0.0)	2 (6.1)	
Other	3 (6.0)	0 (0.0)	3 (9.1)	
**Country of Birth**				0.798
Australia, NZ, Pacific Islanders	35 (70.0)	11 (64.7)	24 (72.7)	
Europe	5 (10.0)	2 (11.8)	3 (9.1)	
South America	4 (8.0)	1 (5.9)	3 (9.1)	
Middle East and Asia	6 (12.0)	3 (17.6)	3 (9.1)	
**Anthropometrics**	**Mean (SD)**	**Mean (SD)**	**Mean (SD)**	
Height (cm)	168.9 (9.6)	179.3 (6.8)	163.6 (5.8)	**<0.001**
Weight (Kg)	70.7 (12.8)	79.6 (9.6)	66.1 (11.9)	**<0.001**
BMI (kg/m^2^)	24.7 (3.5)	24.7 (2.4)	24.6 (3.9)	0.915
Waist Circumference (cm)	86.9 (11.2)	88.9 (8.7)	85.9 (12.3)	0.364
**Weight Status Categories ^†^**	***n* (%)**	***n* (%)**	***n* (%)**	
Underweight	1 (2.0)	1 (3.0)	0 (0)	0.649
Normal Weight	25 (50.0)	16 (48.5)	9 (52.9)	
Overweight	22 (44.0)	14 (42.4)	8 (47.1)	
Obese	2 (4.0)	2 (6.1)	0 (0)	
**Waist Circumference Categories ^‡^**				
Normal	25 (50.0)	13 (39.4)	12 (70.6)	0.105
High Risk	8 (16.0)	6 (18.2)	2 (11.8)	
Very High Risk	17 (34.0)	14 (42.4)	3 (17.6)	
**Hemodynamic Indices**				
*Peripheral Blood Pressure*	**Mean (SD)**	**Mean (SD)**	**Mean (SD)**	
Peripheral SBP (mmHg)	120.0 (13.4)	121.7 (9.1)	119.1 (15.2)	0.454
Peripheral DBP (mmHg)	69.9 (8.4)	69.7 (8.9)	70.0 (8.3)	0.904
**Peripheral Blood Pressure Categories ^§^**	***n* (%)**	***n* (%)**	***n* (%)**	
Normal Blood Pressure	27 (54.0)	20 (60.6)	7 (41.2)	0.399
Elevated Blood Pressure	9 (18.0)	4 (12.1)	5 (29.4)	
Hypertension Stage 1	10 (20.0)	6 (18.2)	4 (23.5)	
Hypertension Stage 2	4 (8.0)	3 (9.1)	1 (5.9)	
***Central Blood Pressure***	**Mean (SD)**	**Mean (SD)**	**Mean (SD)**	
*Central Aortic SBP* (mmHg)	106.8 (13.3)	106.9 (8.5)	106.8 (15.3)	0.971
*Central Aortic DBP* (mmHg)	70.6 (8.7)	70.3 (9.1)	70.8 (9.7)	0.843
Pulse Pressure (mmHg)	36.0 (8.9)	36.2 (7.9)	35.9 (9.5)	0.930
Heart Rate (bpm)	61.5 (10.2)	58.1 (8.9)	63.2 (10.4)	0.092
***Systemic Arterial Stiffness***				
Augmented Pressure (mmHg)	6.8 (6.8)	4.8 (4.2)	7.8 (7.6)	0.077
Augmented Index (%)	16.6 (14.9)	12.2 (9.4)	18.9 (16.8)	0.077
Pulse Wave Velocity (m/s)	9.5 (1.4)	9.5 (1.3)	9.5 (1.5)	0.933

* *p*-values were derived from the Student’s *t*-test for continuous variables and from the chi-square test for categorical variables. Results in bold indicate *p* < 0.05, and are therefore statistically significant. BMI, body mass index; BP, blood pressure; SBP, systolic blood pressure; DBP, diastolic blood pressure; ^†^: Weight status categories: Underweight, BMI < 18.5 Kg/m^2^; Normal weight, 18.5 ≤ BMI < 25 Kg/m^2^; Overweight, 25 ≤ BMI < 30 Kg/m^2^; Obese, BMI ≥ 30 Kg/m^2^. ^‡^: Waist circumference categories: Normal, WC < 80 cm in women and <94 cm in men; High risk, 80–88 cm in women and 94–102 cm in men; Very high risk: WC > 88 cm in women and >102 cm in men. ^§^: Peripheral BP categories: Normal BP, SBP < 120 and DBP < 80 mmHg; Elevated BP, SBP > 120–129.9 and DBP < 80 mmHg; Hypertension Stage 1, SBP 130–139.9 or DBP 80–89.9 mmHg; Hypertension Stage 2, SBP > = 140 or DBP > = 90.

**Table 2:** Effect of low polyphenol OO vs. high polyphenol OO on mean changes in dietary energy, macro- and micronutrient intake.

	Baseline Mean (SEM)	Follow Up Mean (SEM)	Mean Change (95% CI) (Time Effect)	*p*-Value (Treatment * Time Effect)
**Energy intake (KJ/day)**				
Low Polyphenol OO (*n* = 50)	8712.8 (328.3)	10518.9 (344.4)	**1806.1 (1075.4 to 2536.8)**	0.940
High Polyphenol OO (*n* = 50)	8892.6 (328.3)	10659.2 (344.4)	**1766.6 (1035.9 to 2497.3)**	
*p*-value (Treatment effect)	0.700	0.774		
**Protein intake (g/day)**				
Low Polyphenol OO (*n* = 50)	102.0 (5.5)	100.7 (5.2)	−1.3 (−14.3 to 11.8)	0.924
High Polyphenol OO (*n* = 50)	97.4 (5.5)	97.0 (5.3)	−0.4 (−13.4 to 12.7)	
*p*-value (Treatment effect)	0.558	0.619		
**Carbohydrates (g/day)**				
Low Polyphenol OO (*n* = 50)	214.8 (10.1)	213.4 (11.1)	−1.5 (−23.5 to 20.6)	0.972
High Polyphenol OO (*n* = 50)	219.9 (10.1)	217.8 (11.1)	−2.0 (−24.0 to 20.0)	
*p*-value (Treatment effect)	0.726	0.776		
**Total fat intake (g/day)**				
Low Polyphenol OO (*n* = 50)	79.9 (4.0)	129.2 (4.5)	**49.3 (41.1 to 57.4)**	0.571
High Polyphenol OO (*n* = 50)	84.3 (4.0)	130.3 (4.5)	**46.0 (37.8 to 54.1)**	
*p*-value (Treatment effect)	0.441	0.870		
**SFA intake (g/day)**				
Low Polyphenol OO (*n* = 50)	27.7 (1.5)	35.1 (1.9)	**7.4 (4.0 to 10.8)**	0.707
High Polyphenol OO (*n* = 50)	28.8 (1.5)	35.3 (1.9)	**6.5 (3.1 to 9.9)**	
*p*-value (Treatment effect)	0.620	0.953		
**MUFA intake (g/day)**				
Low Polyphenol OO (*n* = 50)	30.6 (1.7)	67.3 (1.9)	**36.8 (33.2 to 40.3)**	0.514
High Polyphenol OO (*n* = 50)	31.8 (1.7)	67.0 (1.9)	**35.1 (31.6 to 38.6)**	
*p*-value (Treatment effect)	0.605	0.877		
**PUFA intake (g/day)**				
Low Polyphenol OO (*n* = 50)	14.6 (1.0)	17.7 (1.0)	**3.1 (1.0 to 5.1)**	0.971
High Polyphenol OO (*n* = 50)	15.7 (1.0)	18.7 (1.0)	**3.0 (1.0 to 5.1)**	
*p*-value (Treatment effect)	0.483	0.469		
**Fibre intake (g/day)**				
Low Polyphenol OO (*n* = 50)	29.7 (1.7)	30.7 (1.8)	0.9 (−3.1 to 4.9)	0.314
High Polyphenol OO (*n* = 50)	29.6 (1.7)	33.5 (1.8)	3.8 (−0.2 to 7.8)	
*p*-value (Treatment effect)	0.963	0.268		
**Sodium intake (mg/day)**				
Low Polyphenol OO (*n* = 50)	2611.1 (269.5)	2287.1 (168.9)	−324.0 (−878.2 to 230.3)	0.994
High Polyphenol OO (*n* = 50)	3096.7 (269.5)	2775.5 (168.9)	−321.2 (−875.5 to 233.1)	
*p*-value (Treatment effect)	0.206	**0.044**		
**Potassium intake (mg/day)**				
Low Polyphenol OO (*n* = 50)	3486,7 (227.0)	3389.3 (170.5)	−97.3 (−631.1 to 436.4)	0.488
High Polyphenol OO (*n* = 50)	3334.4 (227.0)	3501.9 (170.5)	167.5 (−366.2 to 701.3)	
*p*-value (Treatment effect)	0.636	0.642		
**Magnesium intake (mg/day)**				
Low Polyphenol OO (*n* = 50)	574.2 (92.9)	446.0 (18.9)	−128.2 (−308.4 to 52.0)	0.271
High Polyphenol OO (*n* = 50)	433.6 (92.9)	447.6 (18.9)	14.0 (−166.2 to 194.2)	
*p*-value (Treatment effect)	0.287	0.953		
**Calcium intake (mg/day)**				
Low Polyphenol OO (*n* = 50)	1005.0 (92.3)	1056.2 (95.6)	51.1 (−205.1 to 307.3)	0.916
High Polyphenol OO (*n* = 50)	977.3 (92.3)	1009.2 (95.6)	31.9 (−224.3 to 288.1)	
*p*-value (Treatment effect)	0.832	0.729		
**Caffeine intake (mg/day)**				
Low Polyphenol OO (*n* = 46)	199.4 (54.1)	182.0 (35.6)	−17.4 (−122.3 to 87.6)	0.612
High Polyphenol OO (*n* = 43)	242.8 (56.0)	186.7 (36.8)	−56.1 (−164.6 to 52.5)	
*p*-value (Treatment effect)	0.578	0.926		

All statistical analyses were adjusted for gender and age. Results in bold indicate statistical significance (*p* < 0.05). OO, olive oil; SFA, saturated fatty acids; MUFA, monounsaturated fatty acids; PUFA, polyunsaturated fatty acids; SEM, standard error of the mean; CI, confidence interval.

**Table 3:** Effect of low polyphenol OO vs. high polyphenol OO on mean changes in anthropometric indices.

	Baseline Mean (SEM)	Follow Up Mean (SEM)	Mean Change (95% CI) (Time Effect)	*p*-Value (Treatment * Time Effect)
**Weight (kg)**				
Low Polyphenol OO (*n* = 50)	70.8 (1.5)	71.2 (1.5)	**0.4 (0.2 to 0.7)**	0.163
High Polyphenol OO (*n* = 50)	70.7 (1.5)	70.9 (1.5)	0.2 (−0.1 to 0.4)	
*p*-value (Treatment * effect)	0.993	0.902		
**Height (cm)**				
Low Polyphenol OO (*n* = 50)	168.9 (0.9)	169.0 (0.9)	0.1 (−0.2 to 0.4)	0.890
High Polyphenol OO (*n* = 50)	168.9 (0.9)	169.0 (0.9)	0.1 (−0.1 to 0.4)	
*p*-value (Treatment effect)	0.974	0.992		
**BMI (kg/m^2^)**				
Low Polyphenol OO (*n* = 50)	24.7 (0.4)	24.8 (0.4)	0.1 (−0.01 to 0.2)	0.305
High Polyphenol OO (*n* = 50)	24.7 (0.4)	24.7 (0.4)	0.02 (−0.1 to 0.1)	
*p*-value (Treatment effect)	0.993	0.897		
**Waist circumference (cm)**				
Low Polyphenol OO (*n* = 50)	87.1 (1.3)	87.4 (1.2)	0.3 (−0.1 to 0.7)	0.501
High Polyphenol OO (*n* = 50)	87.1 (1.3)	87.3 (1.2)	0.1 (−0.2 to 0.5)	
*p*-value (Treatment effect)	1.000	0.919		

All statistical analyses were adjusted for gender and age. Results in bold indicate statistical significance (*p* < 0.05). OO, olive oil; SEM, standard error of the mean; CI, confidence interval.

**Table 4:** Effect of low polyphenol OO vs. high polyphenol OO on mean changes in hemodynamic and arterial stiffness indices.

	Baseline Mean (SEM)	Follow Up Mean (SEM)	Mean Change (95% CI) (Time Effect)	*p*-Value (Treatment * Time Effect)
**Pulse pressure (mmHg)**				
Low Polyphenol OO (*n* = 50)	35.7 (1.0)	36.4 (1.0)	0.7 (−0.8 to 2.1)	0.296
High Polyphenol OO (*n* = 50)	36.3 (1.0)	35.9 (1.0)	−0.4 (−1.9 to 1.1)	
*p*-value (Treatment effect)	0.653	0.723		
**Pulse rate (bpm)**				
Low Polyphenol OO (*n* = 50)	61.1 (1.3)	59.4 (1.4)	−1.7 (−3.9 to 0.4)	0.403
High Polyphenol OO (*n* = 50)	61.0 (1.3)	60.6 (1.4)	−0.4 (−2.6 to 1.7)	
*p*-value (Treatment effect)	0.954	0.553		
**Augmented pressure (mmHg)**				
Low Polyphenol OO (*n* = 50)	6.5 (0.7)	6.0 (0.7)	−0.5 (−1.6 to 0.6)	0.987
High Polyphenol OO (*n* = 50)	6.9 (0.7)	6.3 (0.7)	−0.5 (−1.6 to 0.5)	
*p*-value (Treatment effect)	0.692	0.714		
**Augmented index (%)**				
Low Polyphenol OO (*n* = 50)	16.2 (1.7)	14.6 (1.8)	−1.7 (−4.2 to 0.8)	0.807
High Polyphenol OO (*n* = 50)	16.6 (1.7)	15.4 (1.8)	−1.2 (−3.7 to 1.3)	
*p*-value (Treatment effect)				
**Pulse wave velocity (m/s)**				
Low Polyphenol OO (*n* = 50)	9.6 (0.1)	9.5 (0.1)	−0.03 (−0.3 to 0.2)	0.926
High Polyphenol OO (*n* = 50)	9.5 (0.1)	9.4 (0.1)	−0.05 (−0.3 to 0.2)	
*p*-value (Treatment effect)	0.679	0.608		

All statistical analyses were adjusted for gender and age. OO, olive oil; SEM, standard error of the mean; CI, confidence interval.

## Supplementary Materials

The following are available online at https://www.mdpi.com/2072-6643/12/8/2272/s1, Table S1: Summary of olive oil volume returned by participants following the two diet interventions.
