# Supplementary material for: The Effect of High Polyphenol Extra Virgin Olive Oil on Blood Pressure and Arterial Stiffness in Healthy Australian Adults: A Randomized, Controlled, Cross-Over Study
Source: Nutrients. 2020 Jul 29;12(8):2272. doi: 10.3390/nu12082272 (PMC7468912; doi:10.3390/nu12082272)
Supplement: Supplementary file 1 [file nutrients-12-02272-s001.pdf]

Table S1. Summary of olive oil volume returned by participants following the two diet interventions.

**1st intervention**	**Low polyphenol OO Mean (SD)**	**High Polyphenol OO Mean (SD)**	***P-value**
Actual remaining OO (ml)	106.4 (152.2)	100.4 (123.6)	0.888
Compliance based on actual remaining (%)	92	92	
**2nd Intervention**			
Actual remaining OO (ml)	105.9 (133.2)	123.1 (164.7)	0.708
Compliance based on actual remaining (%)	92	90	

*P-values derived from the non-parametric Mann-Whitney test. OO, olive oil; SD, Standard Deviation.
